# Protein tyrosine phosphatase PTPN22 regulates IL‐1β dependent Th17 responses by modulating dectin‐1 signaling in mice

**DOI:** 10.1002/eji.201747092

**Published:** 2017-10-20

**Authors:** Harriet A Purvis, Fiona Clarke, Christine K Jordan, Cristina Sanchez Blanco, Georgina H Cornish, Xuezhi Dai, David J Rawlings, Rose Zamoyska, Andrew P Cope

**Affiliations:** ^1^ Academic Department of Rheumatology Centre for Inflammation Biology and Cancer Immunology Faculty of Life Sciences and Medicine King's College London London UK; ^2^ Seattle Children's Research Institute and Departments of Pediatrics and Immunology University of Washington School of Medicine Seattle Washington USA; ^3^ Institute of Immunology and Infection Research Centre for Immunity, Infection and Evolution University of Edinburgh Edinburgh UK

**Keywords:** Autoimmunity, Dectin‐1, IL‐1β, IL‐17, PTPN22

## Abstract

A single nucleotide polymorphism within the *PTPN22* gene is a strong genetic risk factor predisposing to the development of multiple autoimmune diseases. PTPN22 regulates Syk and Src family kinases downstream of immuno‐receptors. Fungal β‐glucan receptor dectin‐1 signals via Syk, and dectin‐1 stimulation induces arthritis in mouse models. We investigated whether PTPN22 regulates dectin‐1 dependent immune responses. Bone marrow derived dendritic cells (BMDCs) generated from C57BL/6 wild type (WT) and *Ptpn22^−/−^* mutant mice, were pulsed with OVA_323‐339_ and the dectin‐1 agonist curdlan and co‐cultured in vitro with OT‐II T‐cells or adoptively transferred into OT‐II mice, and T‐cell responses were determined by immunoassay. Dectin‐1 activated *Ptpn22^−/−^* BMDCs enhanced T‐cell secretion of IL‐17 in vitro and in vivo in an IL‐1β dependent manner. Immunoblotting revealed that compared to WT, dectin‐1 activated *Ptpn22^−/−^* BMDCs displayed enhanced Syk and Erk phosphorylation. Dectin‐1 activation of BMDCs expressing *Ptpn22^R619W^* (the mouse orthologue of human *PTPN22^R620W^*) also resulted in increased IL‐1β secretion and T‐cell dependent IL‐17 responses, indicating that in the context of dectin‐1 *Ptpn22^R619W^* operates as a loss‐of‐function variant. These findings highlight PTPN22 as a novel regulator of dectin‐1 signals, providing a link between genetically conferred perturbations of innate receptor signaling and the risk of autoimmune disease.

## Introduction

The nonsynonymous *PTPN22* polymorphism C1858T (encoding R620W) is a strong risk factor for the development of multiple autoimmune diseases, including rheumatoid arthritis (RA), type I diabetes, lupus and juvenile idiopathic arthritis (JIA) 1. *PTPN22* encodes a tyrosine phosphatase that negatively regulates Src and Syk family kinase (SFK) activity downstream of the T‐cell antigen receptor (TCR) 2. Notably, T‐cells from *Ptpn22* ^−/−^ mice exhibit enhanced TCR signaling resulting in homeostatic expansion of CD4^+^effector cells 3. It has become clear that PTPN22 regulates many pathways in different cell types including the B‐cell receptor 4, the αLβ2 integrin LFA‐1 5 and Toll‐Like Receptor (TLR) signaling pathways 6, 7, 8, 9. While it has become widely accepted that the autoimmune associated *PTPN22* ^R620W^ variant displays reduced binding to the tyrosine kinase Csk, due to a missense mutation in the P1 domain, 2, 10 precisely how the R620W variant affects PTPN22 function is more complex. Both gain‐ and loss‐of‐phosphatase function effects have been reported, depending on the cellular context and signaling pathway under investigation 5, 9, 10, 11.

Antigen presenting cells (APCs) are critical for sensing and mediating effective clearance of pathogens. Ptpn22 is highly expressed in myeloid cells and a functional role for Ptpn22 in regulating TLR signaling pathways in dendritic cells (DC) has been established 9, 12. For example, Ptpn22 negatively regulates LPS‐induced TLR4 signaling resulting in enhanced IL‐12p40 secretion and T‐cell proliferation 9, while TLR3, TLR4 and TLR7 induced type 1 interferon production are positively regulated by the phosphatase through direct binding to TRAF3 leading to TRAF3 ubiquitination and degradation 6, 8. Other studies have demonstrated regulation of inducible phosphorylation of NLRP3, a component of the inflammasome, by Ptpn22 13. These data raise the possibility that Ptpn22 plays a more fundamental role in APC function and the regulation of adaptive immunity than was hitherto appreciated.

C‐type lectin receptor dectin‐1 binds β‐1,3‐glucan, a component of fungal, bacterial and plant cell walls 14. Dectin‐1 engagement regulates antigen uptake, pathogen sensing and inflammatory responses, and promotes DC maturation, a process marked by enhanced expression of cell surface co‐stimulatory molecules and secretion of IL‐1β, IL‐6, IL‐12 and TNFα 15, 16. These functions are mediated by activation of Syk, Erk, MAPK and NFκB 17. Furthermore, dectin‐1 induced DC maturation instructs CD4^+^ T‐cell priming and differentiation into IL‐17 producing T‐helper cells 18, 19. IL‐17 is essential for driving host defense to fungal pathogens, mediating neutrophil recruitment and anti‐microbial peptide production 18. At the same time, IL‐17 has been implicated as a key cytokine in inflammatory responses associated with RA, JIA, and psoriasis 20.

Negative regulation of dectin‐1 signaling is not well understood. Given recent studies demonstrating that Ptpn22 regulates multiple TLR responses in DCs, and that dectin‐1 signaling utilizes the Ptpn22 substrate Syk, we reasoned that Ptpn22 might regulate dectin‐1 signaling, controlling the capability of dectin‐1 matured BMDCs to promote adaptive immune responses.

## Results

### PTPN22 regulates IL‐17 production induced by curdlan activated BMDCs in vitro

**Figure 1:**
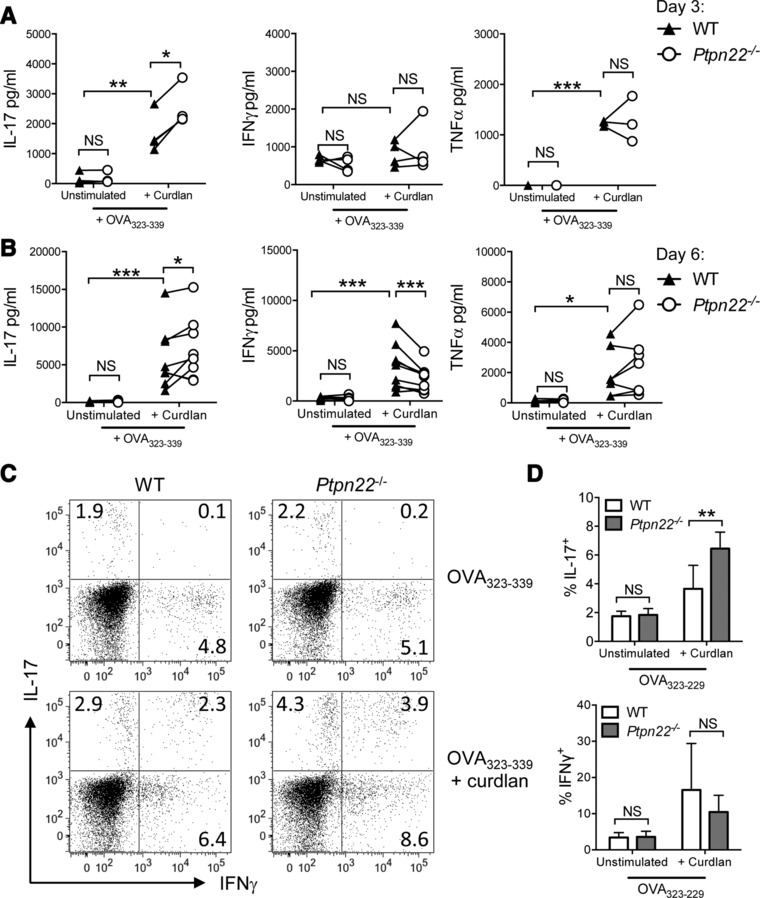
PTPN22 regulates T‐cell dependent IL‐17 responses induced by curdlan stimulated BMDCs in vitro. Wild type (WT) and *Ptpn22* ^−/−^ derived bone marrow derived dendritic cells (BMDCs) were pulsed overnight with OVA_323‐339_ in the presence or absence of curdlan and co‐cultured with OT‐II T‐cells. Cell‐free supernatants were assessed for IL‐17, IFNγ, and TNFα production by immunoassay on day 3 (A) and day 6 (B). Each point represents independent WT (closed triangle) or *Ptpn22* ^−/−^ (open circle) BMDC preparations, connecting lines between WT and *Ptpn22^−/−^* BMDC samples are paired by the same WT OT‐II T‐cell preparation. Data are of (A) 4 and (B) 7 independent experiments with one sample per group per experiment. NS = not significant, *p < 0.05, **p < 0.01, ***p < 0.001 by two‐way ANOVA, applying Sidak's multiple comparisons test. (C and D) T‐cells co‐cultured for 6 days with WT or *Ptpn22* ^−/−^ BMDCs were harvested and replated in IL‐2 and IL‐23 for a further 4 days. At day 10 cells were restimulated for 6 h with PMA and ionomycin in the presence of monensin and intracellular expression of IL‐17 and IFNγ determined by flow cytometry. (C) One representative cytometric dot plot of 5 independent experiments. (D) Pooled data of 5 independent experiments with one sample per group per experiment and represent mean + S.E.M. NS = not significant, **p < 0.01 by two‐way ANOVA, applying Sidak's multiple comparisons test.

We hypothesized that Ptpn22, a negative regulator of Syk, operates in DCs to negatively regulate dectin‐1 signals, and that the absence of Ptpn22 would potentiate DC dependent induction of T‐cell IL‐17 responses. We first compared T‐helper cell differentiation after activation by WT or *Ptpn22* ^−/−^ BMDCs stimulated with the β‐1‐3‐glucan curdlan, a dectin‐1 specific agonist. In vitro WT and *Ptpn22^−/−^* BMDCs, generated in the presence of GM‐CSF, showed no differences in the proportion or number of CD11c^+^ BMDCs generated (Supporting Information Fig. 1A and B). Immature BMDCs were pulsed overnight with OVA_323‐339_ peptide in the presence or absence of curdlan, and co‐cultured with CD4^+^ OT‐II T‐cells. Supernatants were assessed for cytokine expression. As early as day 3 of co‐culture, curdlan stimulated *Ptpn22^−/−^* BMDCs pulsed with OVA_323‐339_ induced significantly more IL‐17 production by T‐cells than WT BMDCs; no differences were observed for IFNγ or TNFα (Fig. 1A). Increased levels of IL‐17 induced by *Ptpn22^−/−^* BMDCs were sustained until day 6 at which point a significant decrease in IFNγ was documented, as compared to WT BMDC:T‐cell co‐cultures (Fig. 1B). Secretion of TNFα was increased by curdlan primed DC, but was not regulated by the presence of PTPN22. This enhanced IL‐17 phenotype was sustained for up to 10 days, as determined by immunoassay (Supporting Information Fig. 1C), and flow cytometry (Fig. 1C and D), but the reduction in IFNγ secretion was lost by this time point. Differences in T‐cell proliferation or viability following co‐culture with WT or *Ptpn22^−/−^* BMDCs did not account for differences in the IL‐17 responses (Supporting Information Fig. 1D and E). These data indicated that *Ptpn22^−/−^* BMDCs have an enhanced capability to induce dectin‐1 dependent IL‐17 T‐cell responses.

### Curdlan activated Ptpn22^−/−^ BMDCs potentiate IL‐17 responses in vivo

**Figure 2:**
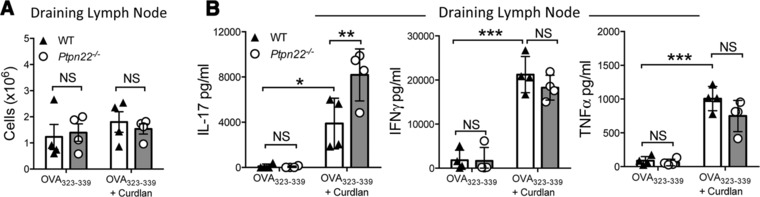
PTPN22 regulates T‐cell dependent IL‐17 responses induced by curdlan stimulated BMDCs in vivo. Wild type (WT) and *Ptpn22* ^−/−^ derived bone marrow derived dendritic cells (BMDCs) were pulsed overnight with OVA_323‐339_ in the presence or absence of curdlan. BMDCs were harvested and injected into the left footpad of OT‐II mice. Seven days post immunization draining popliteal lymph nodes were isolated and the number of cells within the draining (A) lymph nodes determined by Trypan blue counting. Total draining (B) lymph node T‐cells were stimulated with immobilized anti‐CD3 for 48 h and cell‐free supernatant assayed for IL‐17, IFNγ and TNFα by immunoassay. Data are representative of three independent experiments with four mice per group per experiment, each data point representing an individual OT‐II mouse lymph node. Bars represent the mean ± S.D. NS = not significant, **p < 0.01, ***p < 0.001 by two‐way ANOVA, applying Sidak's multiple comparisons test.

To confirm this finding in vivo, WT or *Ptpn22^−/−^* BMDCs pulsed overnight with OVA_323‐339_ in the presence or absence of curdlan, were adoptively transferred into the left footpad of OT‐II mice. After 7 days, draining (left) and non‐draining (right) lymph nodes (LN) of recipient OT‐II mice were harvested for analysis. The number of draining or non‐draining LN cells derived from recipient OT‐II mice was equal in mice that had received either WT or *Ptpn22^−/−^* BMDCs (Fig. 2A, Supporting Information Fig. 2A). Levels of IL‐17, IFNγ, and TNFα were undetectable following stimulation of non‐draining LN T‐cells with anti‐CD3 alone (Supporting Information Fig. 2B). By comparison, stimulation of draining LN T‐cells revealed enhanced levels of IL‐17, IFNγ, and TNFα secretion in recipients of BMDCs pulsed with OVA_323‐339_, and were further enhanced by the BMDCs pulsed with OVA_323‐339_ and curdlan (Fig. 2B). Once again, OVA_323‐339_ and curdlan primed *Ptpn22^−/−^* BMDCs induced significantly more IL‐17 secretion from draining LN than recipients of WT BMDCs (Fig. 2B), while no differences in IFNγ or TNFα secretion were observed. These data validated the in vitro findings in Fig. 1, further confirming that PTPN22 regulates curdlan induced IL‐17 responses in the context of the OT‐II TCR transgenic mouse model.

### Ptpn22 regulates curdlan dependent IL‐1β production by BMDCs

**Figure 3:**
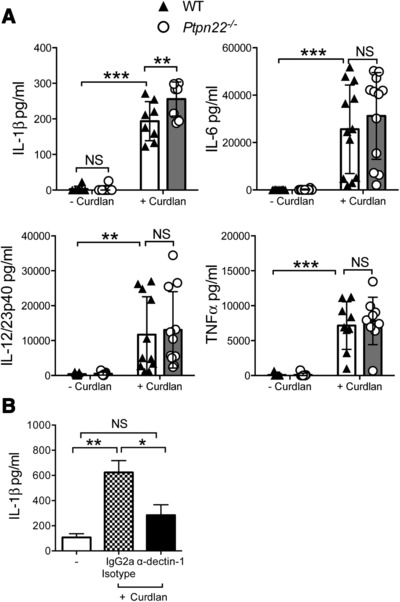
Ptpn22 regulates BMDC IL‐1β secretion in response to curdlan. **(A)** WT and *Ptpn22^−/^* ^−^ derived bone marrow derived dendritic cells (BMDCs) were stimulated for 24 h in the presence or absence of curdlan. Cell‐free supernatants were assessed for IL‐1β, IL‐6, IL‐12/23p40, and TNFα by immunoassay. Data are pooled from 8 to 12 independent experiments with one sample per group per experiment, and represent mean ± S.D. NS = not significant, **p < 0.01, ***p < 0.001 applying two‐way ANOVA, with Sidak's multiple comparisons test. (**B**) WT BMDCs were incubated in the presence or absence of α‐dectin‐1 or IgG2a isotype control antibody for 30 min prior to stimulation for 24 h in the presence or absence of curdlan. Cell‐free supernatants were assessed for IL‐1β by immunoassay. Data are pooled from five independent experiments with one sample per group per experiment, and represent mean + S.E.M. NS = not significant, *p < 0.05, **p < 0.01 applying one‐way ANOVA, with Holm‐Sidak's multiple comparisons test.

Dectin‐1 engagement on BMDCs leads to the production of pro‐inflammatory cytokines such as IL‐1β, IL‐6, IL‐12/23p40 and TNFα, cytokines required for the differentiation of IL‐17 producing T‐cells 16. We compared the cytokine profile of supernatants from WT and *Ptpn22* ^−/−^ BMDCs stimulated in vitro with curdlan. We observed no significant differences in IL‐6, IL‐12/23p40, or TNFα production between WT and *Ptpn22* ^−/−^ BMDCs in response to curdlan (Fig. 3A). However, a modest but significant increase in the secretion of IL‐1β by *Ptpn22* ^−/−^ BMDCs was detected over a range of curdlan concentrations (Fig. 3A and Supporting Information Fig. 3A), a finding that was reproduced when BMDCs were stimulated with another dectin‐1 agonist, heat‐killed *C. albicans* (HKCA) (Supporting Information Fig. 3B). Ptpn22 did not regulate inflammatory cytokine production in response to TLR4 agonist LPS (Supporting Information Fig. 3C). Secretion of IL‐1β was dectin‐1 dependent (Fig. 3B) and dectin‐1 expression was similar between WT and *Ptpn22* ^−/−^ BMDCs (Supporting Information Fig. 4A and B) and was independent of changes in the numbers or proportions of CD11c^+^ BMDCs, or to differences in cell viability (Supporting Information Fig. 1A, B and Supporting Information Fig. 4C). Dectin‐1 signaling also regulates DC maturation and phagocytosis, but we were unable to detect differences between genotypes (Supporting Information Fig. 4D and E). These data demonstrated that Ptpn22 confers a highly selective role in regulating dectin‐1 induced IL‐1β secretion.

### Inhibition of IL‐1β abrogates Ptpn22^−/−^ induced enhancement in IL‐17 secretion

**Figure 4:**
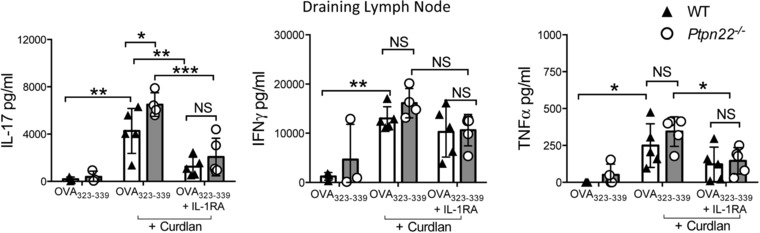
Curdlan stimulated *Ptpn22* ^−/−^ BMDCs induce enhanced IL‐17 responses in an IL‐1β dependent manner. WT and *Ptpn22* ^−/−^ derived bone marrow derived dendritic cells (BMDCs) were pulsed overnight with OVA_323‐339_ in the presence or absence of curdlan. BMDCs were harvested and injected into the left footpad of OT‐II mice in the presence or absence IL‐1RA. 7 days post immunization the draining (left) popliteal lymph nodes were isolated. Total draining lymph node T‐cells were stimulated with immobilised anti‐CD3 for 48 h and cell‐free supernatants assayed for IL‐17, IFNγ and TNFα by immunoassay. Data are representative of two independent experiments with 3–5 mice per group per experiment, each point representing an individual OT‐II mouse lymph node. Data represent mean ± S.D. NS = not significant, *p < 0.05, **p < 0.01, ***p < 0.001 by two‐way ANOVA, applying Sidak's multiple comparisons test.

IL‐1β is a potent cytokine, whose secretion and activity is tightly controlled such that even modest changes in IL‐1β expression may confer functional effects 21. To determine whether differences in IL‐1β secretion between WT and *Ptpn22* ^−/−^ BMDCs were functionally relevant, BMDC:T‐cell co‐culture experiments were repeated in the presence of IL‐1 receptor antagonist (IL‐1RA), a natural ligand which binds to IL‐1R and blocks IL‐1β signaling. In vitro curdlan activated BMDCs induced IL‐17 responses in an IL‐1β dependent manner (Supporting Information Fig. 5A). Furthermore, adoptive transfer of OVA_323‐339_ and curdlan pulsed *Ptpn22* ^−/−^ BMDCs into OT‐II mice by footpad injection resulted in significantly enhanced IL‐17 secretion compared to WT BMDCs, and addition of IL‐1RA mediated a striking reduction in IL‐17 expression, abrogating the difference between WT and *Ptpn22* ^−/−^ BMDC induced IL‐17 expression (Fig. 4, Supporting Information Fig. 5B). In contrast to IL‐17, IL‐1RA mediated reductions in IFNγ and TNFα were more modest. These data suggested that differences in IL‐1β production between WT and *Ptpn22^−/−^* BMDCs are sufficient to mediate functional changes in T‐cell responses.

### Ptpn22 regulates dectin‐1 dependent Syk and Erk activation in BMDCs

**Figure 5:**
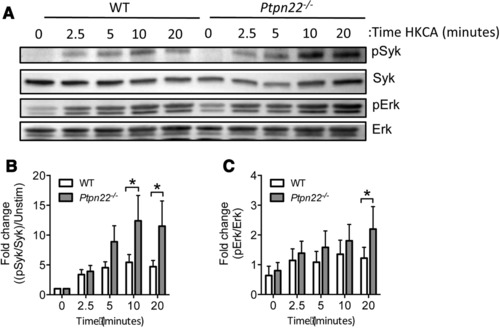
Ptpn22 regulates dectin‐1 dependent Syk and Erk activation in BMDCs. (A–C) WT and *Ptpn22* ^−/−^ BMDCs were stimulated with heat killed *C. albicans* (HKCA) for the indicated time points. Whole cell lysates were resolved by SDS‐PAGE and immunoblotted using specific antibodies to pSyk (Tyr525/526) or Syk, and pErk1/2 (p42/44) or Erk. Quantification using ImageJ software of HKCA induced band intensity measurements pooled from five independent experiments are shown for pSyk (B) and pErk (C), relative to Syk and Erk, respectively. Phosphorylated Syk protein values were normalized to total protein and the fold change to 0 min calculated. Data are mean + S.E.M. from five independent experiments with one sample per group per experiment. *p < 0.05 applying two‐way ANOVA, with Sidak's multiple comparisons test.

Syk activation is crucial for dectin‐1 signaling, raising the possibility that Ptpn22 negatively regulates dectin‐1 induced secretion of IL‐1β via a Syk dependent mechanism. We confirmed previous experimental findings 22, 23 that curdlan induced IL‐1β secretion was Syk and Erk dependent (Supporting Information Fig. 6A–C) using specific Syk and Erk kinase inhibitors. We next compared the kinetics of Syk phosphorylation in WT and *Ptpn22* ^−/−^ BMDCs in response to a dectin‐1 agonist. For technical reasons, including high antibody background signals obtained by immunoblotting when cells were stimulated with curdlan, we used HKCA, a potent stimulator of IL‐1β secretion and dectin‐1 dependent Syk activation (Supporting Information S3B + S6D). Dectin‐1 induced Syk activation was enhanced and prolonged in *Ptpn22* ^−/−^ BMDCs when compared to WT cells (Fig. 5A and B). Following receptor proximal Syk activation, the dectin‐1 signaling pathway diverges, leading to activation of a number of signaling pathways including Erk1/2, NFκB and p38 MAPK. We evaluated each of these pathways in turn and observed that, unlike IκBα and p38, Erk phosphorylation was significantly enhanced in the absence of *Ptpn22* following dectin‐1 stimulation (Supporting Information Fig. 6E, F and Fig. 5A and C). Erk phosphorylation was also significantly increased in curdlan stimulated *Ptpn22* ^−/−^ BMDCs when compared to WT cells (Supporting Information Fig. 6G, H). Together these data indicated that Ptpn22 negatively regulates dectin‐1 induced Syk and Erk phosphorylation.

### The autoimmune risk variant Ptpn22^R619W^ promotes curdlan dependent signaling and IL‐17 production

**Figure 6:**
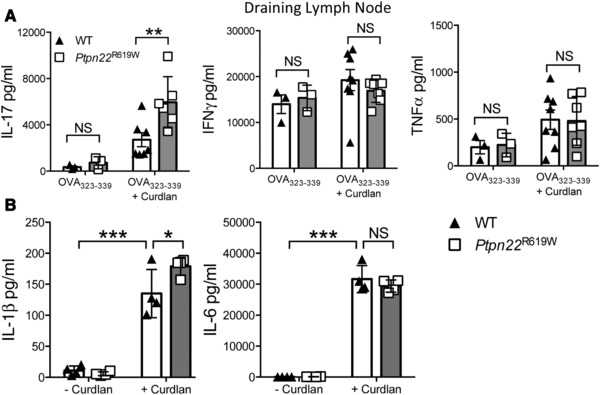
*Ptpn22* ^R619W^ regulates T cell dependent IL‐17 responses induced by curdlan stimulated BMDCs in vivo. WT and *Ptpn22* ^R619W^ derived bone marrow derived dendritic cells (BMDCs) were pulsed overnight with OVA_323‐339_ in the presence or absence of curdlan. BMDCs were harvested and injected into the left footpad of OT‐II mice. Seven days post immunisation the non‐draining and draining popliteal lymph nodes were isolated. Total draining lymph node T‐cells were stimulated with immobilised anti‐CD3 for 48 h and cell‐free supernatant assayed for (A) IL‐17, IFNγ and TNFα by immunoassay. Data are pooled from two independent experiments with 3–4 mice per group per experiment, each data point representing an individual OT‐II mouse lymph node. Bars represent the mean ± S.D. NS = not significant, **p < 0.01 by two‐way ANOVA, applying Sidak's multiple comparisons test. (B) WT and *Ptpn22* ^R619W^ GM‐CSF derived BMDCs were stimulated for 24 h in the presence or absence of curdlan. Supernatants were assessed for secretion of IL‐1β and IL‐6 by immunoassay. Data shown are mean ± S.D. Data are pooled from 4 independent experiments with one sample per group per experiment. NS = not significant, *p < 0.05, ***p < 0.001 by two‐way ANOVA, applying Sidak's multiple comparisons test.

Finally, we investigated whether the mouse orthologue of the human autoimmune disease associated *PTPN22* ^R620W^ variant perturbs dectin‐1 induced BMDC function. BMDCs from WT mice and mice expressing *Ptpn22* ^R619W^ were pulsed with OVA_323‐339_ peptide in the presence or absence of curdlan, and injected into the left footpad of OT‐II recipient mice. After 7 days, draining and non‐draining LN suspensions were prepared prior to stimulation with immobilised anti‐CD3, and assayed for IL‐17, IFNγ and TNFα. As with *Ptpn22* ^−/−^ BMDCs, we observed an increase in the secretion of IL‐17 from draining LN of mice that received *Ptpn22* ^R619W^ BMDCs when compared to WT (Fig. 6A), accompanied by enhanced IL‐1β secretion (Fig. 6B). These data suggest that in the context of dectin‐1 signaling the disease‐associated variant confers reduced function leading to enhanced IL‐1β secretion and increased IL‐17 responses by T‐cells.

## Discussion

We report that in BMDCs derived from both *Ptpn22^−/−^* and *Ptpn22^R619W^* mice dectin‐1 induced Syk and Erk signaling is potentiated, leading to increased IL‐1β secretion and IL‐17 T‐cell responses. These data provide the first association between DC anti‐fungal dectin‐1 signals and the phosphatase Ptpn22, providing evidence supporting a role for this phosphatase as a negative regulator of this signaling pathway.

We found that absence of Ptpn22 increases BMDC secretion of IL‐1β in response to dectin‐1 agonists, potentiating IL‐1β dependent IL‐17 responses in vitro and in vivo. Recent studies have also suggested that Ptpn22 may regulate DC function through IL‐1β dependent mechanisms. For example, in a dextran sulphate induced colitis model, *Ptpn22* ^−/−^ mice developed severe colitis characterised by increased IL‐1β derived from M1 macrophages 24. A further association with IL‐1β was made in the K/BxN serum transfer arthritis model where poly(I:C) administration failed to protect against arthritis in either *Ptpn22* ^−/−^ or *Ptpn22* ^R619W^ mice in part due to potentiated synovial IL‐1β 8. Another study reported that loss of Ptpn22 expression by shRNA in THP‐1 cells or Ptpn22^−/−^ BMDCs reduced secretion of IL‐1β in response to pre‐treatment with LPS followed by activation with NLRP3 activators 13; this is in contrast to our data where LPS treatment alone did not alter IL‐1β secretion (Supporting Information Fig. 3C). Regardless of the specific context, the data point to a conserved function of PTPN22 in regulating IL‐1β expression in myeloid cells. Our data provide an additional link between regulation of dectin‐1 signaling by Ptpn22, IL‐1β secretion and the differentiation of T‐cells to produce IL‐17.

Pathways implicated in negative regulation of dectin‐1 signaling are much less well described than those that activate this signaling cascade. To date several phosphatases have been associated with dectin‐1 signaling. SHIP‐1 is so far the only phosphatase known to directly bind to the dectin‐1 hemi‐ITAM domain and regulate ROS production 25. In contrast, the membrane associated phosphatases CD45 and CD148 may regulate this pathway and there is evidence that they are excluded from the dectin‐1 phagocytic synapse allowing receptor mediated phagocytosis and anti‐microbial responses 26. Here, we report that *Ptpn22* deficiency results in enhanced activation of dectin‐1 signaling intermediates Syk and Erk. Whether Ptpn22 mediates negative regulation through direct interactions with Syk or via indirect effects on other kinases required for initiating Syk activation remains to be determined. In T‐cells, Ptpn22 associates directly with the Syk family kinase ZAP‐70, and loss of Ptpn22 leads to enhanced Erk signaling 5, 27. Erk activation following dectin‐1 engagement requires Syk, H‐Ras, and Card‐9 dependent signals, 22 inducing the secretion of IL‐1β, IL‐6 and TNFα. Our studies also revealed that loss of Ptpn22 expression, or altered function in the setting of the risk variant, conferred a selective increase in dectin‐1 induced IL‐1β. The reasons for this specificity are unclear. One explanation is that there may exist a requirement for distinct Erk signaling thresholds for the induction of certain cytokine signatures 28. We observed a subtle increase in IL‐1β transcription in *Ptpn22^−/−^* BMDCs compared to WT following curdlan stimulation (Supporting Information Fig. 7). IL‐1β processing can be induced by caspase 8 following Syk dependent dectin‐1 signaling, so it is conceivable that differences in activation of caspase 8, which regulates IL‐1β processing, could explain the discrepancy between IL‐1β protein and mRNA expression observed between genotypes 29. An alternate explanation therefore is that Syk and Erk regulate IL‐1β post‐translational processing, as well as transcriptional activity, through regulation of caspase 8 activity.

The prevalence of the *Ptpn22* ^R620W^ polymorphism in the healthy population has led to the theory that it may confer a protective or survival benefit against specific pathogens, such as *M. tuberculosis* 12, a bacterium that induces dectin‐1 dependent IL‐1β 30. Although the association of *Ptpn22* genetic variants with autoimmune disease has been proposed to be due to its role in lymphocyte signaling, environmental factors are also critical to the initiation of autoimmune arthritis. Animal models of arthritis clearly suggest that, besides curdlan, fungal phylotypes within the intestinal microbiota are capable of triggering arthritic autoimmunity 31, 32. Additionally, Sakaguchi et al. demonstrated that subclinical fungal infections drive inflammatory signals leading to spontaneous arthritis in SKG mice, which is also IL‐1β and IL‐17 dependent 33, 34. Recent evidence suggests that vimentin, a cytoskeletal protein secreted by activated cells, is an endogenous ligand of dectin‐1 35. Serum autoantibodies against citrullinated vimentin, common in RA patients, have also been shown to promote osteoclastogenesis and bone resorption in a mouse model 36, raising the possibility that PTPN22 could regulate vimentin‐dectin‐1 driven uptake and presentation of autoantigens, in addition to cytokine secretion. Thus, genetic polymorphisms perturbing DC pathogen sensing may contribute to autoimmunity through a number of distinct mechanisms.

## Materials and methods

### Mice

Wild type (WT) C57BL/6, *Ptpn22* ^−/−^, *Ptpn22* ^R619W^ mice, OT‐II, and OT‐II x Ly5.2 were housed under specific pathogen free (SPF) conditions and used in experiments according to UK Home Office approved protocols. *Ptpn22* ^−/−^ mice and *Ptpn22* ^R619W^ mutant mice were backcrossed for more than 12 generations to the C57BL/6 strain and their generation, genotype and phenotype has been previously described in detail 4, 37. Age‐ and gender‐matched mice were used in the study.

### Bone marrow derived dendritic cell (BMDC) culture

Bone marrow was flushed from femurs and tibias of WT, *Ptpn22* ^−/−^ or *Ptpn22^R619W^* mice by using RPMI‐1640 with L‐glutamine (Corning) containing 1% FBS and penicillin/streptomycin (100 μg/ml). Cells were seeded at 1.5 × 10^6^ cells/ml in 24‐well tissue culture plates in RPMI‐1640 with L‐glutamine supplemented with 10% heat‐inactivated FBS, β‐mercaptoethanol (50 μM), penicillin/streptomycin (100 μg/mL), containing 1% murine GM‐CSF. GM‐CSF was produced from the B78H1/GMCSF.1 cell line. BMDCs were cultured for 6 days at 37°C and 5% CO_2_ and medium replaced on days 3 and 4. At day 6 BMDCs were used in functional assays.

### CD4^+^ T‐cell isolation and BMDC co‐culture

MACS negative selection kit (Miltenyi Biotech) was used to isolate CD4^+^ T‐cells from the lymph nodes (LN) and spleens of 8–16 week old OT‐II mice. T‐cells (2 × 10^7^ cells/mL) were labeled with 2 μM CellTrace Violet (CTV) (Invitrogen) for 20 min at 37°C. BMDCs were pulsed overnight with OVA_323‐339_ peptide (50nM Invivogen) in the presence or absence of curdlan‐AL (100 μg/mL Invivogen). After washing, BMDCs were co‐cultured with CTV labeled CD4^+^ T‐cells at 1:2 BMDC:T‐cell ratio (1 × 10^5^ BMDC:2 × 10^5^ T‐cells) in round bottomed 96‐well plates. After 6 days cells were washed, and replated in RPMI‐1640 with L‐glutamine supplemented with 10% heat‐inactivated FBS, β‐mercaptoethanol (50 μM), penicillin/streptomycin (100 μg/mL), the presence of IL‐2 (1ng/mL Proleukin) and IL‐23 (10 ng/mL R & D Systems) for 4 days.

### Adoptive transfer

WT, *Ptpn22* ^−/−^, or *Ptpn22* ^R619W^ BMDCs were incubated overnight with 50nM OVA_323‐339_ peptide in the presence or absence of curdlan‐AL (100 μg/mL). Cells were harvested and resuspended in PBS prior to injecting 5 × 10^5^ cells in 20 μL volume into the left footpad of OT‐II recipient mice. Where indicated, IL‐1 receptor antagonist (rhIL‐1RA Biolegend) was added to BMDCs immediately before injection (0.5 μg/injection). After 7 days popliteal LNs were isolated, and cell suspensions prepared. Total LN cells were added to anti‐CD3 (1 μg/mL clone; 17A2 Biolegend) coated 96‐well plates. After 48 h cell‐free supernatants were collected and total LN cytokine secretion determined by immunoassay.

### BMDC phenotype

Day 6 BMDCs were stimulated for 24 h in the presence or absence of curdlan‐AL (100 μg/mL). BMDCs were harvested and stained for anti‐mouse CD11c‐PECy7 (clone; N418 Biolegend), MHCcII I‐A^b^‐FITC (clone; AF6‐120.1 Biolegend), CD40‐APC (clone; 3/23 Biolegend), and CD86‐Brilliant Violet 650 (clone; GL‐1 Biolegend) and fixable viability dye eFluor‐506 (eBioscience) in PBS contain anti‐mouse CD16/CD32 (Biolegend). Expression of maturation markers was determined by gating on live, singlet, CD11c^+^ cells and gates were set using fluorescence minus one controls. Cells were fixed in FACS buffer (PBS, 5% FBS, 0.01% NaN_3_) containing 1% PFA and acquired using a Becton Dickinson Fortessa flow cytometer and data analyzed using FlowJo Version 8.7.

### Cytokine immunoassays and cell phenotyping

BMDC cultures were washed and replated at 1 × 10^6^ cells/mL in RPMI‐1640 with L‐glutamine supplemented with 10% heat‐inactivated FBS, β‐mercaptoethanol (50 μM), penicillin/streptomycin (100 μg/mL) and restimulated in the presence or absence of curdlan‐AL (100 μg/mL), LPS (100 ng/mL Invivogen) or heat‐killed Candida albican cells (HKCA 6.25 × 10^5^c/mL strain ATCC 10231 Invivogen) for 24 h. Where indicated anti‐mouse dectin‐1‐IgG (10 μg/mL clone; R1‐8g7 Invivogen), Syk inhibitor II (2 μM Calbiochem), U0126 (10 μM Cell Signaling Technologies) were added 30 min prior to stimulation. Syk inhibitor II is a cell permeable pyrimidine‐carboxamide, which is a potent, selective, reversible and ATP‐competitive inhibitor of Syk 38. U0126 is a highly selective inhibitor of MEK1/2, which induce the activation of Erk 39. IL‐1β, IL‐6, IL‐12/23p40, and TNFα cytokine concentrations were determined in cell‐free supernatants by specific immunoassay. Co‐culture supernatants were harvested at the indicated times. IL‐17, IFNγ, and TNFα were determined by immunoassay. Capture and detection (biotin‐conjugated) antibody pairs were purchased from Biolegend. Cytokine levels were determined using DELFIA‐streptavidin‐europium and DELFIA‐enhancement solution (both Perkin Elmer) and detected on a Victor 1420 multilabel counter (Perkin Elmer). Day 10 BMDCs and OT‐II T‐cell co‐cultures were stimulated with PMA (10 ng/mL), ionomycin (500 ng/mL) and monensin (Biolegend) for 6 h. Cells were stained with anti‐CD3ε‐FITC (clone; 145.2C11 Biolegend), anti‐CD4‐PerCP (clone; RM4‐5 Biolegend) and fixable viability dye eFluor‐506 (eBioscience). Cells were fixed and permeabilised (Foxp3 staining buffer set eBioscience), and incubated with anti‐IL‐17‐AlexaFluor‐647 (clone; TC11‐18H10.1 Biolegend), anti‐IFNγ‐PE (clone; XMG1.2 Biolegend) and anti‐TNFα‐PECy7 (clone; MP6‐XT22 Biolegend) at room temperature for 45 min, washed and resuspended in FACS buffer. Cytokine producing T‐cell populations were determined gating on live, singlet, CD3^+^, CD4^+^ cells and cytokine quadrant gates were set using monensin only controls. Cells were acquired using Becton Dickinson Fortessa or FACSCanto II flow cytometers and data analyzed using FlowJo Version 8.7.

### Heat killed *Candida albicans* uptake

Heat killed *C.albicans* (HKCA) (InvivoGen) were stained with Zombie‐Ultra Violet (UV) dead cell discrimination dye (Biolegend), and washed in PBS at 13 000 rpm for 5 min. BMDCs (2 × 10^5^ cells) were incubated with UV^+^ HKCA (2 × 10^6^) on ice and unbound HKCA was washed off with cold FACS buffer (PBS + 5% FBS + 0.01% NaN_3_) and moved into the 37°C waterbath for time‐points up to 1 h. Subsequently cells were chilled on ice and BMDCs were washed FACS buffer and stained with CD11c‐PE/Cy7 (Biolegend) in PBS containing anti‐CD16/CD32 and fixed with 1% PFA and cells were acquired using a Becton Dickinson Fortessa flow cytometer and data were analyzed using FlowJo software.

### Immunoblotting

BMDCs were washed and resuspended at 6 × 10^6^cells/mL in RPMI‐1640 with L‐glutamine supplemented with β‐mercaptoethanol (50 μM), penicillin/streptomycin (100 μg/mL) for 3 h prior to stimulation with HKCA (6.25 × 10^5^cells/mL) or curdlan (100 μg/mL) at 37°C for 0–20 min. Cells were lysed (1% Triton, 120 mM NaCl, 50 mM Tris, 0.1% SDS, 1 mM EDTA, containing protease/phosphatase inhibitors) and resolved in SDS‐PAGE gels and transferred to PVDF membranes, blocked (Tris, 5% BSA, 0.05% Tween20) and probed with the indicated antibodies; pSyk (clone; C87C1), Syk (clone; D1I5Q), pErk and Erk (mAb Rabbit IgG), Pp38 (clone; D3F9), p38 (clone; D13E1), IκBα (clone; 44D4), pIκBα (clone; 14D4) (all immunoblotting antibodies from Cell Signaling Technologies) followed by anti‐rabbit‐HRP (Dako) secondary antibody. Proteins visualized by SuperSignal chemiluminescent reaction (Pierce) in a ChemiDoc station (BioRad). Densitometry measurements were performed with ImageJ software.

### Real‐time PCR

Total RNA was extracted from BMDCs using TRIzol reagent, and cDNA was reverse transcribed using first strand cDNA synthesis using random hexamers. Gene expression was measured by TaqMan quantitative real‐time PCR using FAM labeled IL‐1β (Mm00434228_m1 Applied Biosystems) and VIC labeled 18S probe. Gene expression was normalized to 18S housekeeper and to unstimulated 0h control.

### Statistical analysis

GraphPad Prism software was used for statistical analysis by one‐way ANOVA with Holm‐Sidak's post‐test or two‐way ANOVA with Sidak's post‐test (paired or unpaired, two‐tails).

## Contributors

H.A.P. performed experiments, analyzed data and wrote the manuscript. F.C., C.K.J. C.S.B., and G.H.C. performed experiments, analyzed data and contributed to the writing of the paper. X.D., D.J.R., and R.Z. developed mouse models and contributed to the writing of the paper. A.P.C. conceived the project, contributed to data analysis and wrote the manuscript. All authors reviewed the manuscript.

## Funding

This research was supported by Arthritis Research UK grants 20218 (awarded to H.A.P and A.P.C), 20525 (awarded to G.H.C, R.Z and A.P.C), Wellcome Trust Investigator Award 096669AIA (awarded to R.Z) and NIH: DP3‐DK097672 and DP3‐DK111802 (to D.J.R). Additional support was provided by the Children's Guild Association Endowed Chair in Pediatric Immunology and the Benaroya Family Gift Fund (to D.J.R.). This work was also supported by infrastructure funded by the National Institute for Health Research (NIHR) BioResource Clinical Research facility and Biomedical Research Centre based at Guy's and St. Thomas’ NHS Foundation Trust and King’s College London (reference: guysbrc‐2012‐17). The content is solely the responsibility of the authors and does not necessarily represent the official views of the National Institutes of Health.

## Conflict of interest

The authors declare no commercial or financial conflict of interest

**Abbreviations**
APC: antigen presenting cell
BMDC: bone marrow derived dendritic cell
CTV: cell trace violet
DC: dendritic cell
HKCA: heat‐killed Candida albicans
IL‐1RA: IL‐1 receptor antagonist
JIA: juvenile idiopathic arthritis
LN: lymph node
OVA: ovalbumin
PTPN22: protein tyrosine phosphatase non‐receptor ‐22
RA: rheumatoid arthritis
S.D: standard deviation
S.E.M: standard error of mean
SFK: Src and Syk family kinase
SPF: specific pathogen free
TCR: T‐cell receptor
TLR: toll‐like receptor
WT: wild‐type

## Supporting information

Peer review correspondence

Supplementary Figures
