# Supplementary material for: Protein tyrosine phosphatase PTPN22 regulates IL‐1β dependent Th17 responses by modulating dectin‐1 signaling in mice
Source: Eur J Immunol. 2017 Oct 20;48(2):306–15. doi: 10.1002/eji.201747092 (PMC5859948; doi:10.1002/eji.201747092)
Supplement: Supplementary file 2 — Supplementary Figures [file EJI-48-306-s002.pdf]

European Journal of Immunology

Supporting Information

for

DOI 10.1002/eji.201747092

Harriet A Purvis, Fiona Clarke, Christine K Jordan, Cristina Sanchez Blanco, Georgina H Cornish, Xuezhi Dai, David J Rawlings, Rose Zamoyska and Andrew P Cope

Protein tyrosine phosphatase PTPN22 regulates IL-1β dependent Th17 responses by modulating dectin-1 signaling in mice

© 2017 WILEY-VCH Verlag GmbH & Co. KGaA, Weinheim

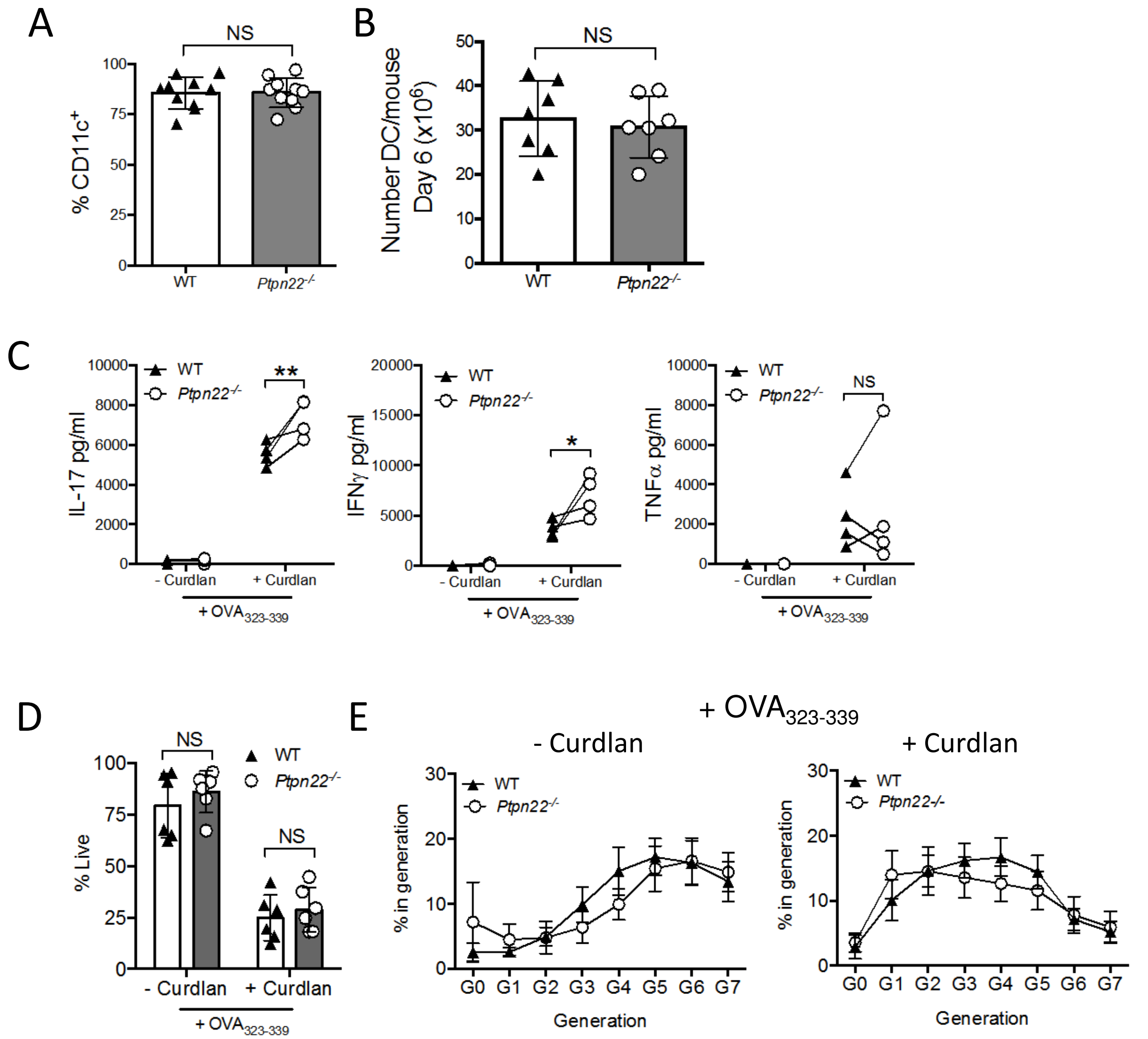

Supplementary Figure 1. Characterisation of WT and *Ptpn22$^{-/-}$* BMDCs co-culture with OT-II T-cells. WT and *Ptpn22$^{-/-}$* GM-CSF derived bone marrow derived dendritic cells (BMDCs) were harvested at day 6 and the proportion **(A)** and number **(B)** of CD11c^{+} BMDCs per 24 well plate determined by cell counting with trypan blue exclusion and flow cytometry. **(A-B)** Data are of >7 independent experiments. Bars represent mean ± s.d. **(C-E)** WT and *Ptpn22$^{-/-}$* BMDCs were pulsed overnight with $OVA_{323-339}$ (50nM) in the presence or absence of curdlan (100 µg/ml) and co-cultured with CTV labelled OT-II T-cells. **(C)** T-cells co-cultured for 6 days with WT or *Ptpn22$^{-/-}$* BMDCs were harvested and replated in IL-2 and IL-23 for a further 4 days. Cell-free supernatants were assessed for IL-17, IFNγ, and TNFα production by immunoassay. Each point represents independent WT (closed symbols) or *Ptpn22$^{-/-}$* (open symbols) BMDCs preparations, each paired with OT-II T-cells; NS = not significant, *p<0.05, **p<0.01 by two-way ANOVA, applying Sidak's multiple comparisons test. At day 6 T-cells were either assessed for viability **(D)** and CTV dilution **(E)** by flow cytometry. **(D)** Data are of 5 independent experiments bars represent mean ± s.d **(E)** data are mean ± s.e.m of 10 independent experiments; NS = not significant.

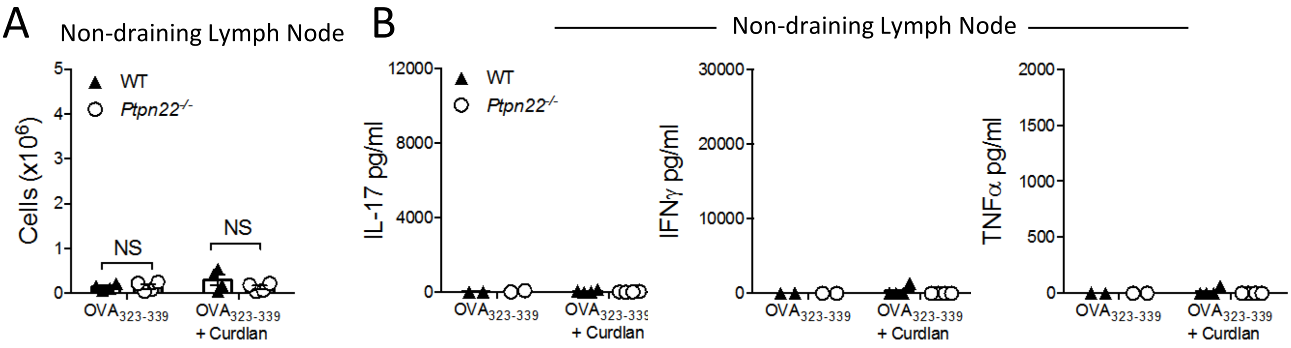

Supplementary Figure 2. PTPN22 regulates T cell dependent IL-17 responses induced by curdlan stimulated BMDCs *in vivo*. Wild type (WT) and *Ptpn22*$^{-/-}$ derived bone marrow derived dendritic cells (BMDCs) were pulsed overnight with $OVA_{323\text{-}339}$ (50nM) in the presence or absence of curdlan (100µg/ml). BMDCs were harvested and injected into the left footpad of OT-II mice. 7 days post immunisation the non-draining and draining popliteal lymph nodes were isolated and the (**A**) number of cells within the non-draining lymph nodes determined. (B) Total non-draining lymph node T-cells were stimulated with immobilised anti-CD3 for 48 hours and cell-free supernatant assayed for IL-17, IFNγ and TNFα by immunoassay. Data are representative of three independent experiments, each data point representing an individual OT-II mouse lymph node. Bars represent the mean ± s.d. NS = not significant.

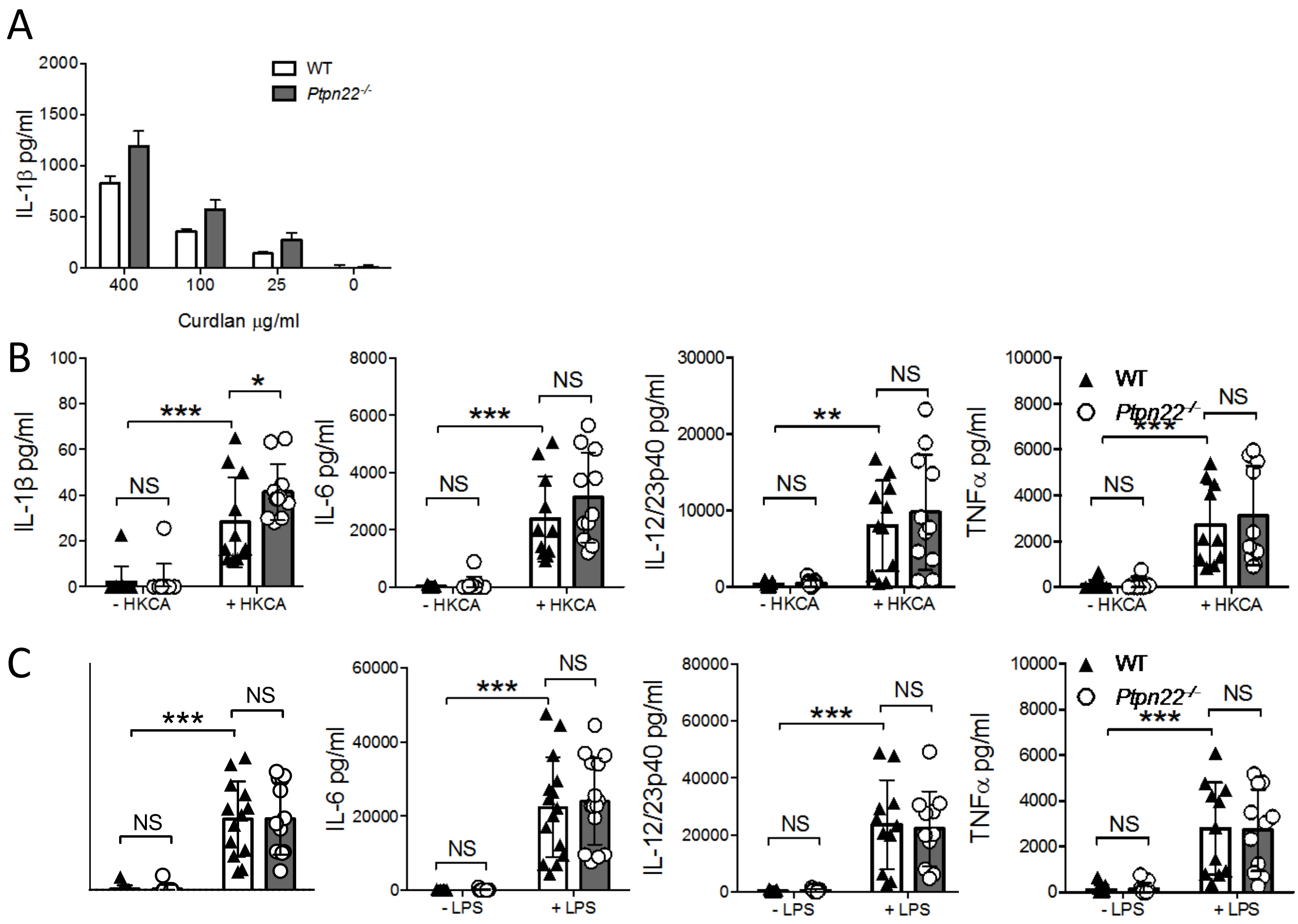

Supplementary Figure 3. WT and *Ptpn22*$^{-/-}$ BMDC activation with curdlan, HKCA and LPS. (A) WT and *Ptpn22*$^{-/-}$ bone marrow derived dendritic cells (BMDCs) (1x10^6 c/ml) were stimulated for 24 hours in the presence or absence of curdlan (400, 100, or 25 µg/ml) and cell-free supernatants were assessed for IL-1β. Data are representative of 3 independent experiments, bars represent mean ± s.d. **(B, C)** WT and *Ptpn22*$^{-/-}$ BMDCs (1x10^6 c/ml) were stimulated for 24 hours in the presence or absence of **(B)** HKCA (6.25x10^5 c/ml) or **(C)** LPS (100 ng/ml). Cell-free supernatants were assessed for IL-1β, IL-6, IL-12/23p40, and TNFα by immunoassay. Data are of >10 independent experiments, bars represent mean ± s.d; NS = not significant, *p<0.05, **p<0.01, ***p<0.001 compared with two-way ANOVA applying Sidak's multiple comparisons test.

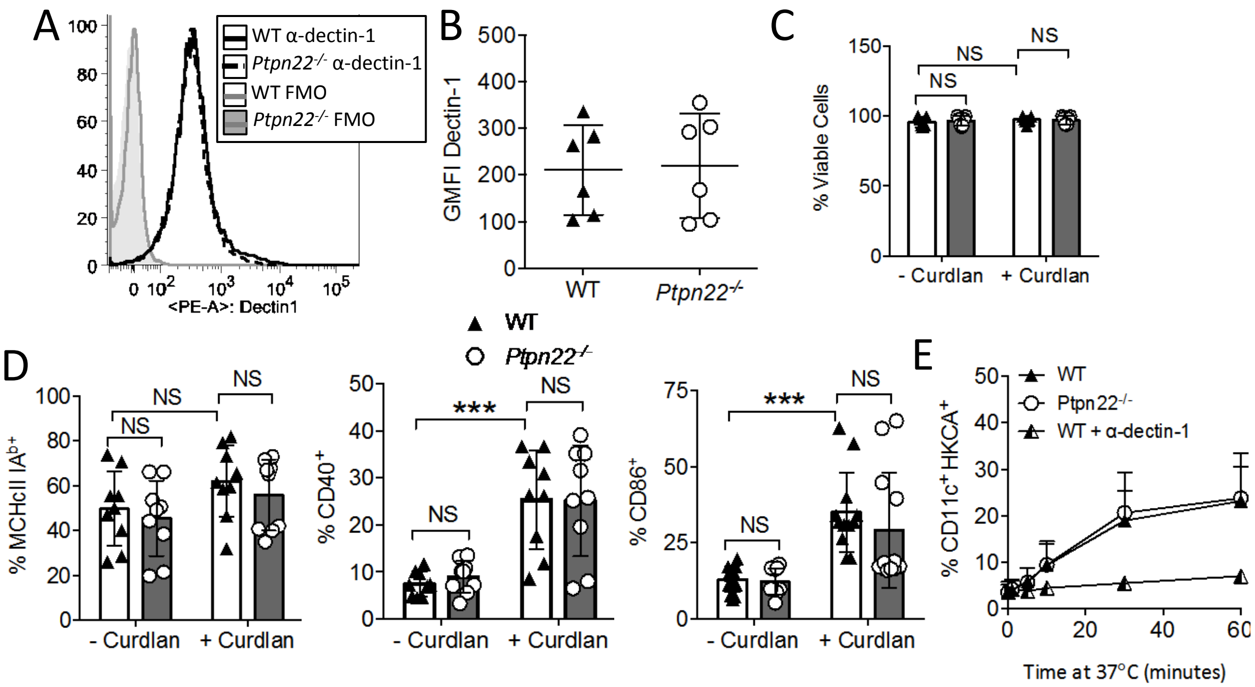

Supplementary Figure 4. Characterisation of WT and *Ptpn22*$^{-/-}$ BMDC activation. (A,B) Day 6 WT and *Ptpn22*$^{-/-}$ BMDCs were surface stained with antibodies towards CD11c and dectin-1 and expression determined by flow cytometry. **(A)** Representative histogram of 1 of 6 independent experiments; solid black line WT; Broken black line *Ptpn22*$^{-/-}$; Grey solid line WT FMO; Grey filled *Ptpn22*$^{-/-}$ FMO **(B)** Dectin-1 geometric mean fluorescence intensity (GMFI) data are of 6 independent experiments; line represents the mean ± s.d. **(C-E)** Day 6 WT and *Ptpn22*$^{-/-}$ BMDCs were stimulated for 24 hours in the presence or absence of curdlan (100µg/ml) and stained for **(C)** viability dye and **(D)** surface markers CD11c, MHCcII IA^b, CD40 and CD86. Cell debris was excluded and the % of viability dye negative cells calculated. Data are of 7-9 independent experiments. Bars represent mean ± s.d. NS = not significant, ***p<0.001 compared with two-way ANOVA, applying Sidak's multiple comparison test. **(E)** WT and *Ptpn22*$^{-/-}$ BMDCs or WT BMDCs pre-treated with anti-dectin-1 (10ug/ml) were incubated at 37℃ with UV labeled HKCA for 0-60 minutes and the percentage of CD11c$^+$UV$^+$ cells determined by flow cytometry. WT and *Ptpn22*$^{-/-}$ data are of 7 independent experiments and WT +α-dectin-1 are of 3 independent experiments. Bars represent mean ± s.d.

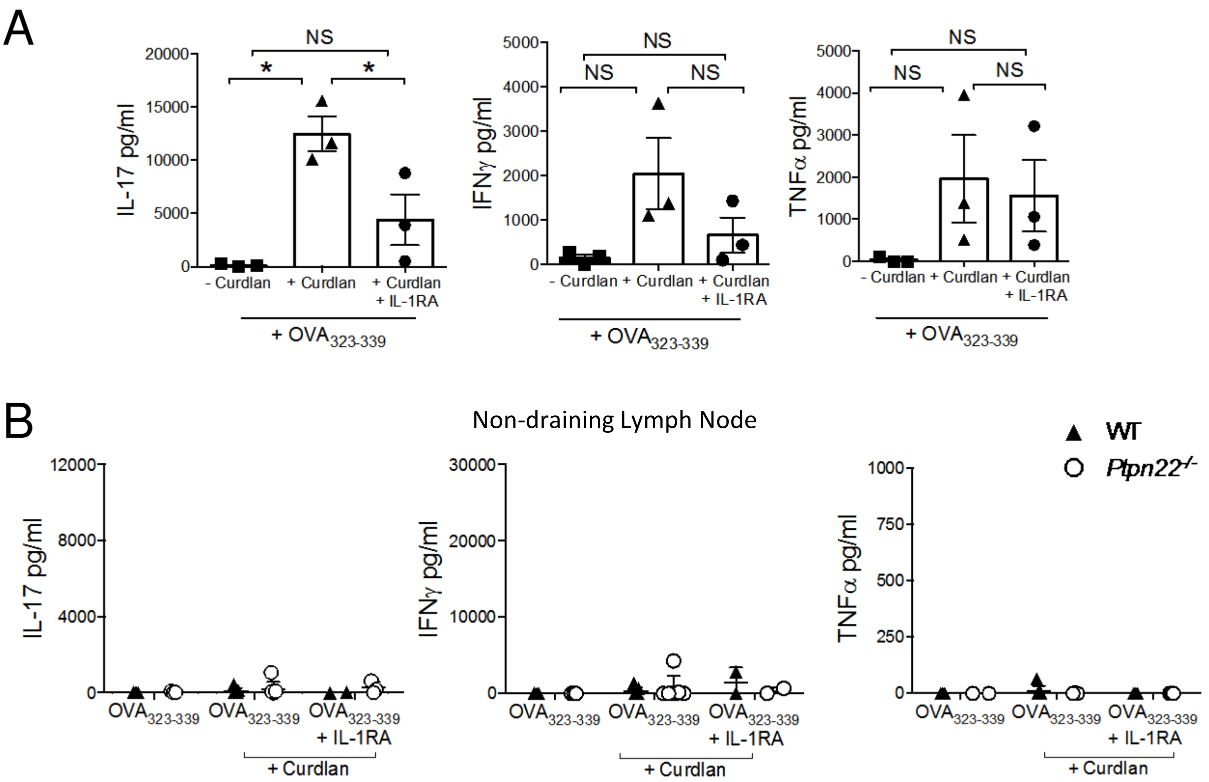

Supplementary Figure 5. Induction of IL-17 by curdlan activated BMDCs is IL-1 dependent. (A) Day 6 bone marrow derived dendritic cells (BMDCs) were pulsed overnight with $OVA_{323-339}$ in the presence or absence of curdlan (100µg/ml). BMDCs were then co-cultured with OT-II T-cells for 6 days in the presence or absence of IL-1RA (0.2µg/ml). Cell-free supernatants were assessed for IL-17, IFNγ, and TNFα by immunoassay. Data show three independent experiments, bars represent mean ± s.d; *p<0.05 compared with one-way ANOVA, with Holm-Sidak's multiple comparisons test. **(B)** WT and *Ptpn22*$^{-/-}$ derived BMDCs were pulsed overnight with $OVA_{323-339}$ (50 nM) in the presence or absence of curdlan (100 µg/ml). BMDCs were harvested and $5x10^5$ cells were injected into the left footpad of OT-II mice in the presence or absence of 0.5mg IL-1RA. 7 days post immunisation the non-draining (right) popliteal lymph nodes were isolated. Total non-draining lymph node T-cells were stimulated with immobilised anti-CD3 for 48 hours and cell-free supernatants assayed for IL-17, IFNγ and TNFα by immunoassay. Data are representative of two independent experiments (N = 3-5 mice per group), each point representing an individual OT-II mouse lymph node. Data represent mean ± s.d. NS = not significant, * p<0.05, **p<0.01, ***p<0.001 by two-way ANOVA, applying Sidak's multiple comparisons test.

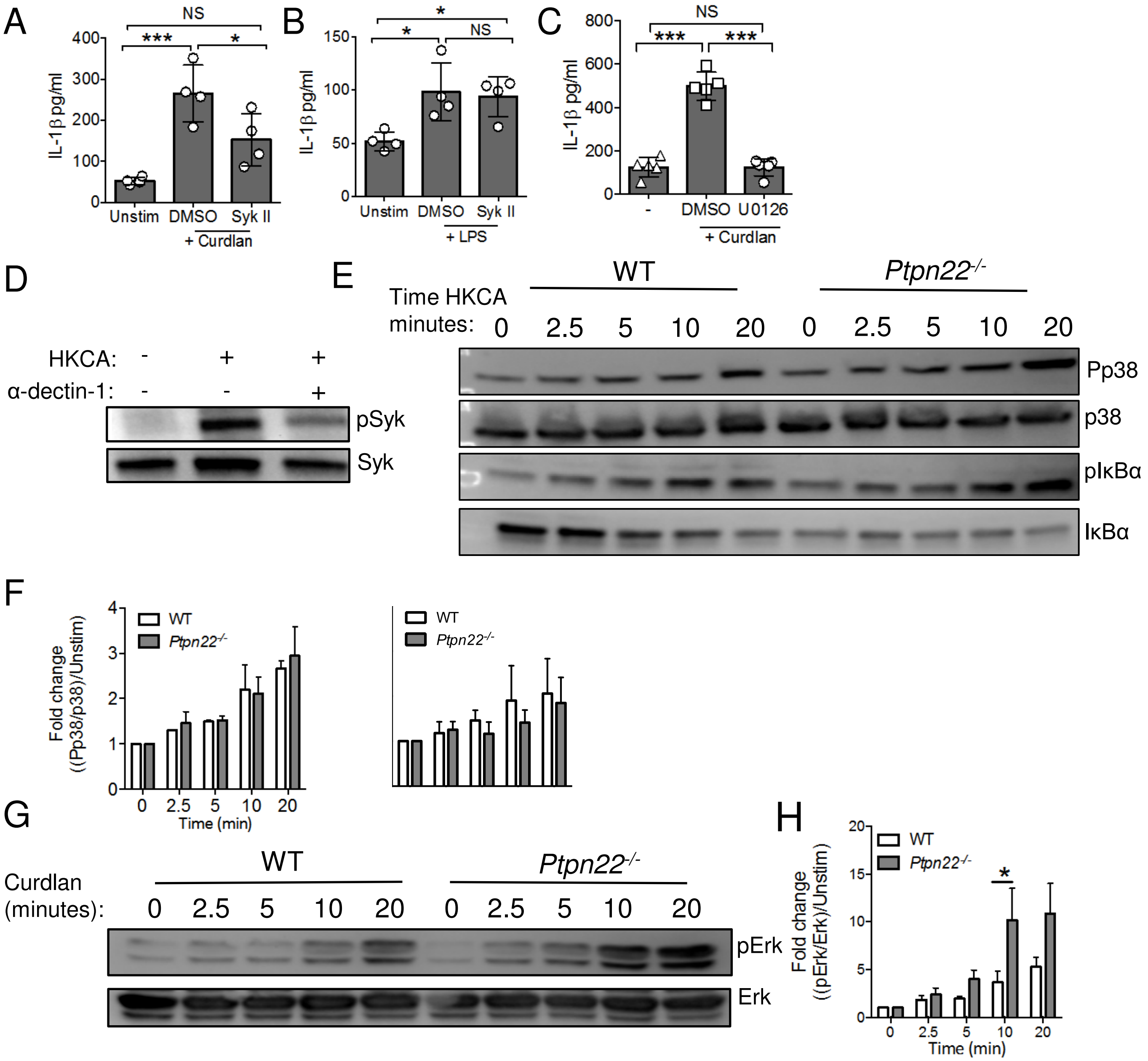

Supplementary Figure 6. Dectin-1 induced WT and *Ptpn22$^{-/-}$* BMDC signalling Bone marrow derived dendritic cells (BMDCs) were pretreated for 30 minutes with (**A, B**) Syk inhibitor SykII (2µM) or (**C**) MEK1/2 inhibitor U0126 (10µM) or DMSO and stimulated for 24 hours in the presence or absence of **(A, C)** curdlan (100 µg/ml) **(B)** LPS 100ng/ml. Cell-free supernatants were assessed for expression of IL-1β by immunoassay. Data are representative of 4-5 independent experiments, presented as mean ± s.d; NS = not significant, **p<0.01; ***p<0.001 applying one-way ANOVA. **(D)** WT BMDCs (3x10^6 c/ml) were pretreated with anti-dectin-1 (5µg/ml) and stimulated for 10 minutes at 37℃ in the presence of HKCA (2.5 x10^6 c/ml). Whole cell lysates were blotted for total and pSyk **(E-F)** WT and *Ptpn22$^{-/-}$* BMDCs (3x10^6 c/ml) were stimulated for 0-20 minutes at 37℃ in the presence of HKCA (6.25 x10^5 c/ml). Whole cell lysates were blotted for total and Pp38 and total and pIκBα. **(E)** Representative blots of 3 independent experiments **(F)** ImageJ quantification of band intensity. Phosphorylated protein values were normalised to total protein and the fold change to 0 min calculated. Bars represent the mean of 3 independent experiments ± s.e.m. **(G-H)** WT and *Ptpn22$^{-/-}$* BMDCs were stimulated for 0-20 minutes at 37℃ in the presence of curdlan (100µg/ml). Whole cell lysates were blotted for total and pErk **(G)** Representative blots of 3 independent experiments **(H)** ImageJ quantification of band intensity. Phosphorylated protein values were normalised to total protein and the fold change to 0 min calculated. Bars represent the mean of 3 independent experiments ± s.e.m.; *p<0.05 compared with two-way ANOVA, applying Sidak's multiple comparison test.

.

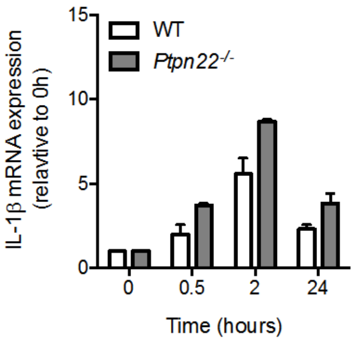

Supplementary Figure 7. Curdlan induced IL-1β trancription. (A) WT and *Ptpn22*$^{-/-}$ bone marrow derived dendritic cells (BMDCs) were stimulated for 0-24h at 37℃ in the presence of curdlan (10 µg/ml). Expression of IL-1β was determined by real-time PCR and normalised to expression of 18S and IL-1β expression at 0h. Bars represent the mean of triplicate values representative of 3 independent experiments ± s.d.
