# Supplementary material for: Protein tyrosine phosphatase PTPN22 regulates IL‐1β dependent Th17 responses by modulating dectin‐1 signaling in mice
Source: Eur J Immunol. 2017 Oct 20;48(2):306–15. doi: 10.1002/eji.201747092 (PMC5859948; doi:10.1002/eji.201747092)
Supplement: Supplementary file 1 — Peer review correspondence [file EJI-48-306-s001.pdf]

European Journal of Immunology

Protein Tyrosine Phosphatase PTPN22 regulates IL-1β dependent Th17 responses by modulating dectin-1 signaling in mice

Harriet A Purvis, Fiona Clarke, Christine K Jordan, Cristina Sanchez Blanco, Georgina H Cornish, Xuezhi Dai, David J Rawlings, Rose Zamoyska and Andrew P Cope

Correspondence: Dr. Harriet A Purvis: Academic Rheumatology, Centre for Inflammation Biology and Cancer Immunology, 1st Floor New Hunts House, Guy's Campus, King's College London, Great Maze Pond, London, United Kingdom

---

Review Timeline:	Submission date:	19-Apr-2017
	First Editorial decision:	26-May-2017
	Revision received:	24-Aug-2017
	Accepted:	18-Sept-2017

Handling Executive Committee member: Prof. Kenneth Murphy

Please note that the correspondence below does not include the standard editorial instructions regarding preparation and submission of revised manuscripts, only the scientific revisions requested and addressed.

First Editorial Decision
26-May-2017

Dear Dr. Purvis,

We apologize for the delay in the peer review of your Manuscript ID eji.201747092 entitled "Protein Tyrosine Phosphatase PTPN22 regulates IL-1β dependent Th17 responses by modulating dectin-1 signaling" which you submitted to the European Journal of Immunology. The manuscript has now been reviewed and the comments of the referees are included at the bottom of this letter.

A revised version of your manuscript that takes into account the comments of the referees will be reconsidered for publication. Should you disagree with any of the referees concerns, you should address

this in your point-by-point response and provide solid scientific reasons for why you will not make the requested changes.

You should also pay close attention to the editorial comments included below. **In particular, please edit your figure legends to follow Journal standards as outlined in the editorial comments. Failure to do this will result in delays in the re-review process.**

Please note that submitting a revision of your manuscript does not guarantee eventual acceptance, and that your revision will be re-reviewed by the referees before a decision is rendered.

If the revision of the paper is expected to take more than three months, please inform the editorial office. Revisions taking longer than six months may be assessed by new referees to ensure the relevance and timeliness of the data.

Once again, thank you for submitting your manuscript to European Journal of Immunology and we look forward to receiving your revision.

Yours sincerely,
Eloho Etemire

On behalf of
Prof. Kenneth Murphy

Dr. Eloho Etemire
Editorial Office
European Journal of Immunology
e-mail: ejied@wiley.com
www.eji-journal.eu

****

Reviewer: 1

Comments to the Author
PTPN22 is an important regulatory component of T cell receptor and plays key role in innate immune receptor signaling. Allelic variants of PTPN22 in humans are linked to several autoimmune syndromes, possibly due to over-inflammatory immune responses. In some mouse models, PTPN22 inhibits T cell

responses, whereas it increases innate immune cell activation and proinflammatory cytokine production. Herein, the authors consider the role of PTPN22 in dectin-1 dependent immune responses. Antigen-specific CD4 T cell responses were compared in vitro and in vivo when activated with dectin-1 activated bone marrow-derived dendritic cells (BMDCs) derived from WT and PTPN22-KO mice. PTPN22-KO BMDCs were more efficient at inducing Th17 differentiation of CD4 T cells via an IL-1b-dependent mechanism. Similar results were obtained when BMDCs derived from PTPN22-R620W knock-in mice. The data are consistent with PTPN22 acting to promote T cell responses that are autoimmune-prone. The manuscript addresses an interesting topic and is well written. Enthusiasm would have been increased had the authors employed an autoimmune model clearly showing that PTPN22-regulated dectin-induced DC->CD4 axis affects disease activity.

Concerns

1. What is the meaning of the connecting lines in Fig. 1? Did you perform paired t test analysis? Were there experiments pooled in any of the Figures? If so, it should be stated in the legend.
2. What are the differences between Fig. 1B and SF.1C with regards the IFN-g data? They seem completely opposite.
3. Please show data collectively for Fig. 1C.
4. Statistics in Fig. 2D (between WT and PTPN22-KO +curdlan) seem overestimated. Are you sure the p value is <0.01? The non-draining lymph node data could be moved to supplement.
5. According to previous research (Wang Y et al, Immunity, 2013) WT and PTPN22-KO DCs differ significantly in activation and proinflammatory cytokine production. How do the authors explain the lack of such differences in their phenotypic analysis (SF. 3)?
6. The data could be strengthened with additional studies using a different TCR transgenic model.
7. The in vivo experiments would be best if performed as follows: adoptive transfer of CFSE labelled OTII (CD45.1+) cells into B6 mice, followed by injection of BMDCs in the footpad.
8. How do the authors explain the lack of differences in IL-1b at the mRNA level (SF6)?

Minor

1. Some data that are in the SFs could be moved to the main Figs, and some that are in the main Figs moved to the supplement. For example, SF2A could be moved to main Figs, whereas F4A could be place in the supplement. Also Fig. 5A is confirmatory and could be moved in the supplement. Instead, SF3 could be moved in the main Figs.
2. The supporting information sometimes results redundant to main Figs and could be reduced.
3. The authors accidentally state that the BMDCs were œstimulated• with OVA323.

Reviewer: 2

Comments to the Author

Figure 1C indicates that the flow profiles of cytokine production are representative of 5 independent experiments. A statistical analysis of compiled data from these 5 experiments should be presented to support the statement that the enhanced IL-17 phenotype was sustained for up to 10 days.

Details of the kinase inhibitors used the study is inadequate. The authors state that "curdlan induced IL-1b secretion was Syk and Erk dependent (Figure 5A,B)." In figure 5 and the figure 5 legend the terms SykII and U0126 are used without noting that U0126 is an Erk inhibitor. In the Methods it notes U1026 (not U0126, which in looking at the Cell Signaling Technologies website is the correct designation) was obtained from Cell Signaling Technologies without detailing that it inhibits both MEK1 and MEK2 (and notes that these enzymes are also called Erk kinases). The description of the Syk inhibitor noted as "Syk inhibitor II" at the Calbiochem website has very little information about its specificity. The authors should provide some basic information about the inhibitors and provide references to studies in the literature defining the specificities of the two inhibitors (no kinase inhibitor is completely specific). To increase confidence in the interpretation of the results of this experiment using the kinase inhibitors, it would be valuable to add a specificity control to the experiments presented in Figures 5A and B showing, for example, that IL-1b induced by LPS is not inhibited by these compounds.

typos noted:

The word "mice" is missing in the following sentence in the Discussion:

A further association with IL-1β was made in the K/BxN serum transfer arthritis model where poly(I:C) administration failed to

protect against arthritis in either Ptpn22-/- or Ptpn22R619W ---mice--- in part due to potentiated synovial IL-1β [8].

There are formatting problems in references 9, 12, 15 and 16.

First Revision – authors' response

24-Aug-2017

Reviewer 1.

We would like to thank the reviewer for their constructive comments and have improved the manuscript according to their suggestions.

1. *What is the meaning of the connecting lines in Fig. 1? Did you perform paired t test analysis? Were there experiments pooled in any of the Figures? If so, it should be stated in the legend.*

The connecting lines in Figure 1 indicate the differences between OT-II T-cell responses induced by WT or *Ptpn22*$^{-/-}$ BMDC when co-cultured with the same preparation OT-II T-cells from WT OT-II mice; the data points are therefore linked. The statistical analysis was performed by paired two-way ANOVA as the data compare two variables; genotype and curdlan response. Each point and connecting line indicates independent BMDC and OT-II T-cell preparations. In response to the reviewer's comments we have revised the Fig. 1 legend to clarify the data and analyses performed.

2. *What are the differences between Fig. 1B and SF.1C with regards the IFN-g data? They seem completely opposite.*

The differences between data in Figure 1B and SF.1C relate to the time points of the cytokine response; Fig. 1B is day 6 and SF.1C is day 10. The reviewer raises an interesting point in that the IFNγ response is opposite between the day 6 and day 10 time points, something we have also found paradoxical. At day 6 the cells are harvested and washed and placed into fresh media. We believe that the differences found at day 10 are due to washing the cells and replating them at day 6 in fresh medium.

3. *Please show data collectively for Fig. 1C.*

We have now included the collective data and statistical analysis for Fig. 1C as Fig. 1D and have updated the figure legend and text accordingly.

4. *Statistics in Fig. 2D (between WT and PTPN22-KO + curdlan) seem overestimated. Are you sure the p value is <0.01? The non-draining lymph node data could be moved to supplement.*

We have repeated the statistical analysis and checked the p value for these data and it is correct p=0.0052. We thank the reviewer for their suggestion of moving the non-draining lymph node data to the supplement along with suggestions to rearrange further sections of data in subsequent comments. We have now included changes in line with the reviewers' suggestions listed below under minor comments.

5. *According to previous research (Wang Y et al, Immunity, 2013) WT and PTPN22-KO DCs differ significantly in activation and proinflammatory cytokine production. How do the authors explain the lack of such differences in their phenotypic analysis (SF. 3)?*

During our detailed analysis of the impact of Ptpn22 on dectin-1 induced DC responses we observed no consistent differences in cell surface maturation markers. Furthermore, we observed no difference in WT vs *Ptpn22*$^{-/-}$ DC maturation following *in vivo* stimulation with heat killed *C.albicans*. Therefore, we do not believe that the data reflect an artefact of using *in vitro* generated BMDC. The effect of Ptpn22 on dectin-1 mediated DC maturation appears to be modest and specific to IL-1β. The reason for this is unclear; one reason may be that the increase in activatory signals observed in the absence of Ptpn22 is not of sufficient strength or duration to confer broader effects on the DC phenotype. An alternate explanation linked to the reviewers comment below, regarding the lack of difference in IL-1β at the mRNA level, is that Ptpn22 is controlling IL-1β secretion at a post-translational level. For example Syk is able to initiate caspase-8 cleavage and in turn the activation of IL-1β. We believe this to be an alternate route by which Ptpn22 could specifically regulate IL-1β secretion rather than conferring more broad effects on cell surface DC maturation as observed in the context of poly I:C stimulation.

6. *The data could be strengthened with additional studies using a different TCR transgenic model.*

We appreciate the reviewers' comment that it would be interesting to see if the IL-17 response observed in our investigations extends beyond the context of OT-II TCR transgenic mouse. The focus of our investigation was on the impact of Ptpn22 on the DC response to dectin-1 and so we have not invested so much time and resources on a detailed investigation of the T-cell response. Although we have not performed these experiments with a different TCR transgenic model we have now included a comment in the results section to address this point (2.2; final sentence), emphasising that the differences in IL-17 response are reported in the context of T cell responses using the OT-II TCR transgenic mouse model.

7. *The in vivo experiments would be best if performed as follows: adoptive transfer of CFSE labelled OTII (CD45.1+) cells into B6 mice, followed by injection of BMDCs in the footpad.*

As suggested by the reviewer we have performed this experiment, transferring OT.II T-cells and either WT or *Ptpn22*$^{-/-}$ BMDC into C57BL/6 mice and then restimulating the popliteal lymph nodes with $OVA_{323-339}$ peptide. As with the experiments performed directly in OT.II mice we observed that IL-17 production was enhanced in mice, which received *Ptpn22*$^{-/-}$ BMDC pulsed with curdlan and $OVA_{323-339}$ peptide (Figure A at the

bottom of letter). We hope that this reassures the reviewer that the data presented are truly representative.

8. *How do the authors explain the lack of differences in IL-1b at the mRNA level (SF6)?*

We too were surprised not to observe differences in IL-1β mRNA levels between WT and *Ptpn22*$^{-/-}$ dectin-1 activated BMDC. However, as mentioned above IL-1β secretion is controlled both at the pre and post-translationally. Caspase 8 can be directly activated by Syk to induce IL-1β activation (see Gringhuis *et al* Nature Immunology 2012). We suspect that Ptpn22 may regulate caspase 8 dependent cleavage and processing of IL-1β.

Minor points
1 and 2. Some data that are in the SFs could be moved to the main Figs, and some that are in the main Figs moved to the supplement. For example, SF2A could be moved to main Figs, whereas F4A could be place in the supplement. Also Fig. 5A is confirmatory and could be moved in the supplement. Instead, SF3 could be moved in the main Figs. The supporting information sometimes results redundant to main Figs and could be reduced.

We thank the reviewer for their suggestion and have made a number of changes to the figure arrangement based on these suggestions (see below). We also appreciate that the reviewer feels that some of the information within the supplement is redundant. In the absence of specific suggestions we do feel, however, that these data may be helpful to readers with different scientific backgrounds and as such have kept all supplementary data originally presented.

- Figure 2A has now been moved to the supplement, becoming Supplementary Figure 2.
- Figure 4A has now been moved to the supplement, becoming Supplementary Figure 5B.
- Figure 5A,B has now been moved to the supplement, becoming Supplementary Figure 6A-C.

3. The authors accidentally state that the BMDCs were “stimulated” with OVA323.

We have amended the relevant statements to say ‘pulsed’ with OVA_{323} rather than ‘stimulated’.

Reviewer: 2

We would like to thank the reviewer for their constructive comments and have improved the manuscript according to their suggestions.

1. Figure 1C indicates that the flow profiles of cytokine production are representative of 5 independent experiments. A statistical analysis of compiled data from these 5 experiments should be presented to support the statement that the enhanced IL-17 phenotype was sustained for up to 10 days.

We have now included the collective data for Fig. 1C as Fig. 1D, including statistical analysis, and have updated the figure legend and text accordingly.

2. Details of the kinase inhibitors used the study is inadequate. The authors state that "curdlan induced IL-1b secretion was Syk and Erk dependent (Figure 5A,B)." In figure 5 and the figure 5 legend the terms SykII and U0126 are used without noting that U0126 is an Erk inhibitor. In the Methods it notes U1026 (not U0126, which in looking at the Cell Signaling Technologies website is the correct designation) was obtained from Cell Signaling Technologies without detailing that it inhibits both MEK1 and MEK2 (and notes that these enzymes are also called Erk kinases). The description of the Syk inhibitor noted as "Syk inhibitor II" at the Calbiochem website has very little information about its specificity. The authors should provide some basic information about the inhibitors and provide references to studies in the literature defining the specificities of the two inhibitors (no kinase inhibitor is completely specific). To increase confidence in the interpretation of the results of this experiment using the kinase inhibitors, it would be valuable to add a specificity control to the experiments presented in Figures 5A and B showing, for example, that IL-1b induced by LPS is not inhibited by these compounds

Data presented in Figure 5 A and B sought to confirm already well-documented findings in the literature demonstrating that dectin-1 induced IL-1β secretion is Syk and Erk dependent. These previous studies demonstrated this using both Syk$^{-/-}$ mice, as well as Syk and Erk inhibitors. We have inserted a sentence in the results section 2.5 along with references to describe these data and to emphasise that these data are confirmatory. As suggested by the reviewer we did perform a specificity control for the Syk II inhibitor and found that the concentration which we had used was at the high end of the dose response curve, and was capable of inhibiting LPS induced IL-1β, most likely due to off-target effects on Src family kinases. We now include data using a lower concentration of the Syk II inhibitor demonstrating inhibition of curdlan induced IL-1β under circumstances where LPS induced IL-1β is preserved (see Figure B at the bottom of this letter). As these data merely confirm previous investigations, reviewer 1 suggested that Figure 5A and B should be moved to the supplement. We have therefore moved these data (including experiments using the lower concentration of the Syk II inhibitor) to Supplementary Figure 6A-C; text and figure legends have been adjusted accordingly. As per reviewer 2’s suggestion the accompanying figure legend

has been modified to note that the Syk II inhibitor and U0126 inhibitors are Syk and MEK1/2 inhibitors. Thank you for pointing out the U0126 typo. We have included further information and references in the methods section describing the inhibitors in more detail.

4. Typos noted:
The word "mice" is missing in the following sentence in the Discussion: A further association with IL-1β was made in the K/BxN serum transfer arthritis model where poly(I:C) administration failed to protect against arthritis in either Ptpn22$^{-/-}$ *or Ptpn22R619W ---mice--- in part due to potentiated synovial IL-1β [8].*

We have inserted the word mice.

5. There are formatting problems in references 9, 12, 15 and 16.

We have rectified the formatting issues within the references as raised by the reviewer. These changes are marked up within the reference list.

Supporting figures in response to reviewer comments

Figure A

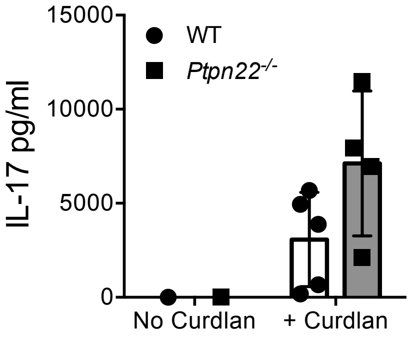

Figure A. C57BL/6 mice received 2.5x10[5] WT OT-II T-cells i.v. The following day C57BL/6 mice received WT or *Ptpn22*$^{-/-}$ derived bone marrow derived dendritic cells (BMDC) were pulsed overnight with $OVA_{323\text{-}339}$ (50 nM) in the presence or absence of curdlan (100 µg/ml) 1 x10[5] into the left footpad. 7 days post immunisation the non-draining (right) popliteal lymph nodes were isolated. Total draining lymph node T-cells were stimulated with $OVA_{323\text{-}339}$ (5 µg/ml) for 48 hours and cell-free supernatants assayed for IL-17 by immunoassay. Data are of one experiment, each point representing an individual C57BL/6 mouse draining lymph node.

Figure B

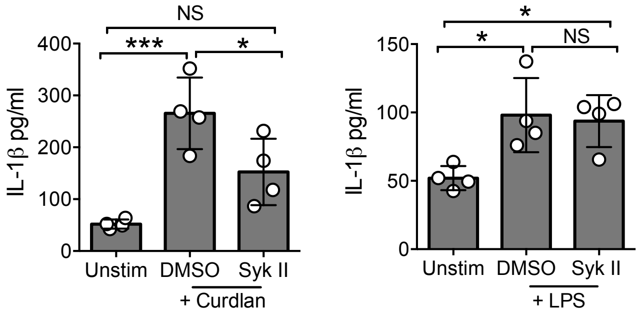

Figure B. Bone marrow derived dendritic cells (BMDC) were pretreated for 30 minutes with **(A, B)** Syk inhibitor SykII (2 µM) DMSO and stimulated for 24 hours in the presence or absence of **(A)** curdlan (100 µg/ml) **(B)** LPS (100 ng/ml). Cell-free supernatants were assessed for expression of IL-1β by immunoassay. Data are representative of 4 independent experiments, presented as mean ± s.d; NS = not significant, *p<0.05; ***p<0.001 applying one-way ANOVA.

European Journal of Immunology

Second Editorial Decision

06-Sep-2017

Dear Dr. Purvis,

It is a pleasure to provisionally accept your manuscript entitled "Protein Tyrosine Phosphatase PTPN22 regulates IL-1β dependent Th17 responses by modulating dectin-1 signaling" for publication in the European Journal of Immunology. For final acceptance, please follow the instructions below and return the requested items as soon as possible as we cannot process your manuscript further until all items listed below are dealt with.

Please note that EJI articles are now published online a few days after final acceptance (see Accepted Articles: http://onlinelibrary.wiley.com/journal/10.1002/(ISSN)1521-4141/accepted). The files used for the Accepted Articles are the final files and information supplied by you in Manuscript Central. You should therefore check that all the information (including author names) is correct as changes will NOT be permitted until the proofs stage.

We look forward to hearing from you and thank you for submitting your manuscript to the European Journal of Immunology.

Yours sincerely,
Eloho Etemire

on behalf of
Prof. Kenneth Murphy

Dr. Eloho Etemire
Editorial Office
European Journal of Immunology
e-mail: ejied@wiley.com
www.eji-journal.eu
